# Impact of Mutations in the Hemagglutinin of H10N7 Viruses Isolated from Seals on Virus Replication in Avian and Human Cells

**DOI:** 10.3390/v10020083

**Published:** 2018-02-14

**Authors:** Anne Dittrich, David Scheibner, Ahmed H. Salaheldin, Jutta Veits, Marcel Gischke, Thomas C. Mettenleiter, Elsayed M. Abdelwhab

**Affiliations:** Institute of Molecular Virology and Cell Biology, Friedrich-Loeffler-Institut, Federal Research Institute for Animal Health, Südufer 10, 17493 Greifswald-Insel Riems, Germany; annedittrich17@yahoo.de (A.D.); david.scheibner@fli.de (D.S.); dr.ahmedhatem@ymail.com (A.H.S.); jutta.veits@fli.de (J.V.); marcel.gischke@fli.de (M.G.); thomas.mettenleiter@fli.de (T.C.M.)

**Keywords:** influenza, H10N7, harbor seals, receptor binding, adaptation, poultry, interspecies transmission

## Abstract

Wild birds are the reservoir for low-pathogenic avian influenza viruses, which are frequently transmitted to domestic birds and occasionally to mammals. In 2014, an H10N7 virus caused severe mortality in harbor seals in northeastern Europe. Although the hemagglutinin (HA) of this virus was closely related to H10 of avian H10N4 virus, it possessed unique nonsynonymous mutations, particularly in the HA1 subunit in or adjacent to the receptor binding domain and proteolytic cleavage site. Here, the impact of these mutations on virus replication was studied in vitro. Using reverse genetics, an avian H10N4 virus was cloned, and nine recombinant viruses carrying one of eight unique mutations or the complete HA from the seal virus were rescued. Receptor binding affinity, replication in avian and mammalian cell cultures, cell-to-cell spread, and HA cleavability of these recombinant viruses were studied. Results show that wild-type recombinant H10N4 virus has high affinity to avian-type sialic acid receptors and no affinity to mammalian-type receptors. The H10N7 virus exhibits dual receptor binding affinity. Interestingly, Q220L (H10 numbering) in the rim of the receptor binding pocket increased the affinity of the H10N4 virus to mammal-type receptors and completely abolished the affinity to avian-type receptors. No remarkable differences in cell-to-cell spread or HA cleavability were observed. All viruses, including the wild-type H10N7 virus, replicated at higher levels in chicken cells than in human cells. These results indicate that H10N7 acquired adaptive mutations (e.g., Q220L) to enhance replication in mammals and retained replication efficiency in the original avian host.

## 1. Introduction

Influenza A virus, a member of the family *Orthomyxoviridae*, possesses an RNA genome of eight gene segments that encodes at least 10 viral proteins and is enclosed in a nucleoprotein wrapped in a lipid bilayer envelope [1]. According to the antigenic properties of two membrane glycoproteins, hemagglutinin (HA) and neuraminidase (NA), 18 HA and 11 NA subtypes are differentiated [2,3]. Each virus displays distinct HA and NA proteins. Except for H17N10 and H18N11, which were isolated from bats [2], all other HA and NA subtypes were isolated from avian species [3]. Wild aquatic birds are the reservoir for all avian influenza viruses (AIVs). Interspecies transmission from wild birds to domestic birds and mammals has been frequently reported [4,5]. It has been estimated that more than 200 bird species are susceptible to AIVs [4]. Mammals, including humans, mink, horses, pigs, raccoons, and aquatic animals, are accidental hosts for AIVs, producing infections from asymptomatic to lethal [1,6].

The HA of influenza viruses is a major determinant of cross-species transmission, virulence, and immunogenicity [7]. HA consists of two polypeptides, the N-terminal HA1 and the C-terminal HA2, that remain connected by a disulfide bridge after the proteolytic activation of HA0 by host cell proteases acting at the cleavage site motif [8]. HA1 contains the receptor binding domain (RBD), which forms a shallow pocket in the head domain and is surrounded by 130-loop, 150-loop, 190-helix, and 220-loop structures. Some mutations in or adjacent to the RBD may modulate the binding affinity of influenza viruses to sialic acid (SA), linked to the sugar galactose in α2,3 orientation in AIVs, or in α2,6 orientation in human influenza viruses [3,6]. Moreover, HA1 possesses five immunogenic epitopes, designated A to E, which are major regions for stimulation of and binding to antibodies. Epitopes A and B are located in the head region adjacent to the pocket formed by the RBD. Mutations in these epitopes result in antigenic drift, enabling the virus to escape from the host immune response [9,10].

Avian influenza H10Nx viruses or antibodies have been detected in mink, walrus, raccoons, pigs, dogs, and humans [11,12,13,14,15,16,17]. In 2014, high mortality of harbor seals (*Phoca vitulina*) in northwestern Europe was associated with infection by H10N7 influenza virus [18,19,20,21,22]. The virus was first reported in seals in Sweden and Denmark, and subsequently spread to seals off the coasts of Germany and the Netherlands [23]. Although the virus had the capacity to replicate in the mammalian respiratory tract, e.g., in ferrets, the observed high mortality was most likely due to secondary bacterial infections [21]. Genetically, the virus was closely related to contemporary viruses isolated from wild and domestic birds in Europe. In particular, HA showed 98–99% identity to A/mallard/Sweden/133546/2011(H10N4) [18,19,20]. Although several unique amino acid substitutions in the HA protein of seal viruses compared to putative avian parental strains have been described [18,19,20], the biological function of these mutations, including increased affinity to mammalian receptors, remains unknown. In this study, we compared the HA protein of H10N7 viruses isolated from seals and birds from Europe. Mutations unique to the seal virus in the HA1 domain were identified and their impact on receptor binding specificity, virus replication in avian and mammalian cells, cell-to-cell spread, and cleavage activation was investigated.

## 2. Materials and Methods

### 2.1. Viruses and Cells

A/seal/Germany/AR2351/1/14(H10N7) was obtained from the virus repository at the Friedrich-Loeffler-Institut and kindly provided by Timm C. Harder. A/turkey/England/384/79(H10N4) was kindly provided by Ian Brown at the Animal and Plant Health Agency, Weybridge, UK. A/PR/8/1934(H1N1) was kindly provided by J. Stech, FLI, and quail H4N2 virus was kindly provided by Beate Crossley, University of California, Davis, CA, USA. Human-embryonic kidney 293T cells (HEK-293T), Madin-Darby canine kidney cells type II (MDCKII), and human lung adenocarcinoma 549 cells (A549) were obtained from the cell-culture collection at FLI. Primary chicken embryo kidney (CEK) cells were prepared according to standard procedures [24].

### 2.2. Sequence Analysis

Sequences of the hemagglutinin of H10N7 viruses isolated from harbor seals in Europe in 2014 and their avian H10Nx counterparts, in addition to those detected in mammals, as well as avian H10Nx viruses from non-European countries were retrieved from the Global Initiative on Sharing Avian Influenza Data (GISAID) and the Influenza Virus Database of the National Center for Biotechnology Information. Acknowledgment of authors and laboratories submitting to the GISAID is provided in Supplementary Table S1. All sequences were aligned with Multiple Alignment using Fast Fourier Transform [25], then visualized and edited by BioEdit 7.1.7 (Ibis Therapeutics, Carlsbad, CA, USA) [26]. Asparagine-linked potential glycosylation was predicted by the motif N-X-S/T, where X can be any amino acid (aa) except proline. The H10-HA numbering in this study excludes the 16-residue signal peptide. Predicted locations of the HA mutations mentioned in this study were imposed on the tertiary structure of the HA protein of H10N4 virus using SWISS-MODEL (http://swissmodel.expasy.org/) and then viewed in Geneious v.8.1.3 (Biomatters Ltd., Auckland, Australia) and edited manually.

### 2.3. Generation of Recombinant Viruses

cDNA of all 8 segments of H10N4 virus and the HA segment of H10N7 virus was generated using a universal primer targeting the conserved 12 nucleotides at the 5’-end as previously published [27]. Each gene segment was amplified using Phusion PCR (New England BioLabs, Frankfurt am Main, Germany) and segment-specific primers (Eurofins, Ebersberg, Germany) [28]. Amplicons were excised and extracted from agarose gels using the QIAquick Gel Extraction Kit (Qiagen, Hilden, Germany). Purified products were cloned in pHWS*ccdB* [28], followed by transformation of competent *E. coli* strain TOP10™ (Invitrogen, Thermo Fisher Scientific, Schwerte, Germany), XL1-Blue™, or SURE2™ (Stratagene Europe, Amsterdam, Netherlands). Plasmids were extracted by Qiagen Plasmid Mini, Midi, or Maxi Kit (Qiagen, Hilden, Germany). DNA concentration was adjusted to about 1 µg/µL. Insertion of indicated mutations in the HA of H10N4 was done by QuikChange II XL Site-Directed Mutagenesis Kit (Agilent Technologies, Waldbronn, Germany). Primers used for generation of mutants are available upon request. Sequences were analyzed to exclude any unwanted mutation by Sanger sequencing using an ABI BigDye Terminator v.1.1 Cycle Sequencing Kit (Applied Biosystems, Langen, Germany).

All recombinant viruses were rescued after transfection of mixed HEK293T and MDCKII cell culture using Lipofectamine^®^ 2000 and Optimum [28]. Viruses were propagated in 9-to-11-day-old specific pathogen-free embryonated chicken eggs. Inoculated eggs were candled daily for survival of embryos. Eggs that contained dead embryos and those that survived for 5 days post-inoculation were chilled at 4 °C before harvesting of the allantoic fluid. Hemagglutination test was done using 1% chicken erythrocytes, and hemagglutinating units were determined as described [29]. Allantoic fluids with an HA titer >2^4^ and bacteria-free as determined on blood agar plates were pooled, aliquoted, and stored at −70 °C. Infectivity titers were determined by plaque assay as described below.

### 2.4. Replication Kinetics

Replication kinetics of recombinant viruses were compared on A549 and CEK cells using 1 plaque-forming unit (PFU) per 1000 cells for 1, 8, 24, and 48 h postinfection (hpi). The cells were infected in the presence of 2 µg/mL trypsin and then incubated at 37 °C or 33 °C with 5% CO_2_. At the indicated time points, cells and supernatants were harvested and stored in cryotubes at −80 °C until use. Virus titers were quantified by plaque assay using MDCKII cells. The assay was conducted in duplicate and repeated 2 to 3 times, and the results are expressed as average and standard deviation of all replicates.

### 2.5. Plaque Test and Cell-to-Cell Spread

Virus was titrated using MDCKII cells in the presence of trypsin using 10-fold serial dilutions in minimum essential medium (MEM). Virus dilutions were added to the cells for 1 h at 37 °C and 5% CO_2_, and then the inocula were removed by absorption of infected medium by vacuum. Cells were washed with 1× PBS (pH 7.4) and then overlaid with semisolid agar containing MEM supplemented with bovine serum albumin (BSA). All plates were incubated at 37 °C and 5% CO_2_ for 3 days, then fixed by formaldehyde containing crystal violet for at least 1 day. Virus titers were expressed as plaque-forming unit per ml (PFU/mL). To investigate the impact of specific mutations on cell-to-cell spread in MDCKII cells in the presence of trypsin, 50 to 100 plaques were measured using Nikon Instruments NIS Elements Basic Research software (version 4.0, Nikon, Duesseldorf, Germany). Results are shown as percentage relative to plaques produced by the wild-type H10N4 virus.

### 2.6. Receptor Binding Specificity Assay

Avian α2,3-SA specificity was determined by solid-phase binding assay [30,31]. Briefly, asialofetuin–horseradish peroxidase (HRP) conjugate was sialylated using CMP-sialic acid (Sigma Aldrich, Steinheim, Germany) and α-2,3-(*N*)-sialyltransferase from *Pasteurella multocida* (Sigma Aldrich, Germany). Twelve well plates were coated with 10 µg/mL fetuin from fetal bovine serum (Sigma Aldrich, Germany). Viruses were adjusted to 5 log_2_ HA units and 50 μL of indicated viruses was added, followed by incubation of plates overnight at 4 °C. Unbound virus was removed by aspiration, and the plates were washed with PBS and blocked by 0.2 mL of PBS containing 2% bovine serum albumin (Sigma Aldrich, Germany) for 1 h at room temperature. Plates were washed with PBS containing TWEEN^®^80 (Sigma Aldrich, Germany). Twofold dilution of 50 µL α2,3-labeled fetuin-HRP was done, and the solution was incubated for 1 h at 4 °C then washed with PBS containing TWEEN^®^80. A total of 100 μL of tetramethylbenzidine substrate was added for 30 min at room temperature, and the reaction was stopped with 50 μL of 50 mM H_2_SO_4_. The optical density was measured at 450 nm using a Tecan ELISA Reader (Tecan, Crailsheim, Germany).

Receptor binding specificity to mammalian α2,6-linked SA was tested using modified turkey erythrocytes [32]. Briefly, SA was removed by incubation of 1% turkey erythrocytes (TRBCs) with Vibrio cholerae neuraminidase (Sigma Aldrich, Germany) [32]. Desialylated Turkey erythrocytes (TRBCs) were suspended in PBS containing 1% BSA. Loss of TRBC hemagglutination activity was confirmed by incubation with human H1N1/PR8 with high affinity to human-like receptors and avian H4N2 with high affinity to avian receptors. TRBCs were resialylated using α2,6-(*N*)-sialyltransferase (Takara ClonTech, Saint-Germain-en-Laye, France) in a final concentration of 1.5 mM CMP-sialic acid (Sigma-Aldrich, Germany). Modified TRBCs were suspended in PBS containing 1% bovine serum albumin to a final concentration of 0.5%. Resialylation was confirmed by hemagglutination using PR8 and H4N2 viruses. The affinity of all recombinant viruses (adjusted to ~10^7^ PFU/mL) to different receptors was compared using standard hemagglutination assay against modified TRBCs, desialylated RBCs, and original turkey RBCs according to the World Organization for Animal Health (OIE) protocol [29]. The assay was run in duplicate and repeated twice. Results are expressed as average of all replicates.

### 2.7. Heat Stability

The stability of indicated viruses was tested using 600 µL aliquots containing 10^5^ PFU of each virus after incubation at 56 °C for different time durations (0, 0.5, 1, 2, 3, and 4 h). The test was conducted in duplicate. The reduction in virus infectivity was determined by plaque assay.

### 2.8. Western Blot Analysis

MDCKII cells were seeded in T25 flasks 1 day before infection. The cells were washed with MEM with 5% bovine serum albumin (BSA) and then infected with virus at a multiplicity of infection of >1 PFU per cell at 37 °C for 1 h. Cells were washed twice with PBS, and MEM with BSA was added with or without trypsin (2 µg/mL) at 37 °C/5% CO_2_. At 6 and 24 hpi, the cells were harvested with cell scrapers and collected in Falcon tubes. The cell suspension was centrifuged at 14,000 rpm for 15 min. The supernatant was aspirated and the cell pellets were washed with PBS. This was repeated twice before suspending cell pellets in Laemmli buffer (Sigma-Aldrich, Germany) and PBS at a ratio of 1:1. Moreover, deglycosylation of indicated viruses was studied using PNGase F (New England BioLabs, Frankfurt am Main, Germany) at 37 °C for 1 h following the vendor’s recommended protocol. All samples were stored at −20 °C until further analysis. Samples were thawed at room temperature, then incubated at 99 °C for 5 min, followed by centrifugation at 14,000 rpm for 5 min. The proteins were separated on 10% or 12% SDS-PAGE along with BenchMark™ Pre-Stained Protein Ladder in SDS-PAGE buffer for 45 min at 200 volts. For the step of transferring the proteins onto a nitrocellulose membrane, a semidry blotting gadget was used at 25 volts for 2 h. Transferring viral proteins to nitrocellulose membranes and processing the blots were done as previously described [33], with little modification. The serum used was obtained 10 days after infection of chickens with H10N4 virus intravenously in a previous experiment. Serum in 0.5% skim milk (1:100) was added and incubated at 37 °C overnight. Then the membrane was washed twice with Tris-Buffered Saline containing Tween (TBST) for 30 s, once for 15 min, then twice for 5 min. Thereafter, blots were incubated with peroxidase-conjugated species-specific secondary antibodies at a dilution of 1:20,000 in TBST for 30 min, followed by several washing steps and continuous shaking. Immunodetection was achieved by chemiluminescence using Supersignal West Pico chemiluminescent substrate kit (Pierce, Thermo Scientific, Rockford, IL, USA). Images were captured using a Bio-Rad VersaDoc imaging system (Bio-Rad, Hercules, CA, USA) and Quantity One software (version 4.6.3, Bio-Rad, Hercules, CA, USA).

### 2.9. Statistics

Variations in replication kinetics in different cells, heat stability, and receptor binding affinity were assessed using ANOVA with post hoc Tukey test. Differences in plaque size were compared using Kruskal-Wallis test and Wilcoxon test with Bonferroni correction. Data were analyzed using R version 2.14.0 from the R Foundation for Statistical Computing, available at the R-project website (http://www.r-project.org), and differences were considered significant at a *p*-value < 0.05.

## 3. Results

### 3.1. Sequence Analysis

**Figure 1:**
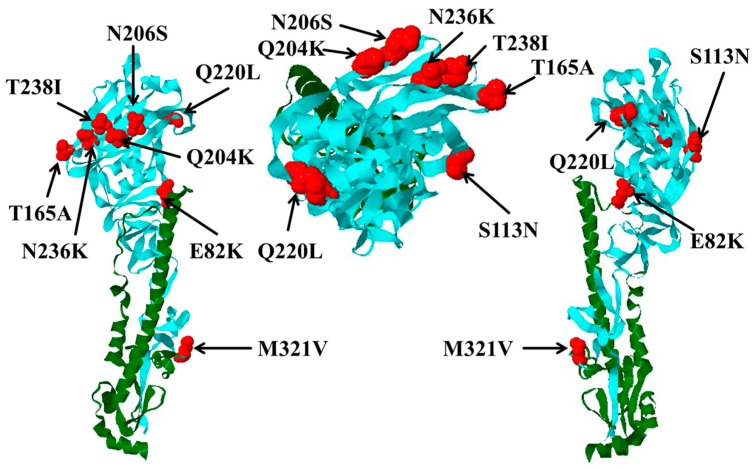
Locations of hemagglutinin 1 (HA1) mutations and their impact on receptor binding activity and cleavability of hemagglutinin. Predicted locations of HA mutations in seal H10N7 viruses compared to avian H10Nx viruses are shown in red. HA1 is shown in cyan and HA2 in green. Left and right views are about 180 degrees apart (e.g., front vs. back views), and head view is in the middle.

**Figure 2:**
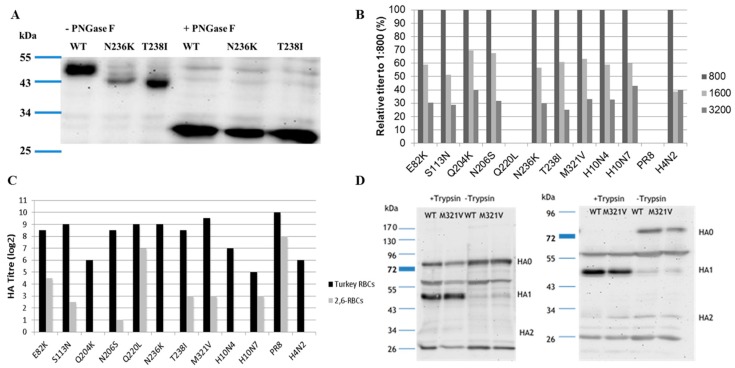
(**A**) Molecular weight of the HA of H10N4 (wild type) compared to viruses carrying N236K or T238I without (-PNGase) or with (+PNGase) treatment using PNGase F; (**B**) affinity of different recombinant H10 viruses to avian-type receptors using different concentrations (1:800; 1:1600, and 1:3200) of 2,3-labelled fetuin analyzed in solid-phase assay; (**C**) receptor binding affinity to turkey erythrocytes expressing both avian and mammalian sialic acid receptors, and modified turkey erythrocytes expressing 2,6-mammalian receptors only. Results are expressed as average of hemagglutination titer of two independent runs, each run in duplicate; (**D**) cleavage of HA of H10N4 compared to H10N4 with M321V substitution in the presence (+Trypsin) or absence (-Trypsin) of trypsin 6 or 24 h after infection of Madin-Darby canine kidney cells type II (MDCKII) using polyclonal antiserum generated 10 days after intravenous inoculation of chickens with H10N4 virus. All viruses were constructed by reverse genetics, except H10N7 wild-type virus, which was used as a control.

Sequences of all European H10Nx viruses from birds and mammals in GenBank and GISAID available to 12, October, 2017 were retrieved and analyzed. A total of 26 seal virus sequences were collected, from Sweden (*n* = 4), Denmark (*n* = 5), Germany (*n* = 11), and the Netherlands (*n* = 6), isolated in 2014 (*n* = 25) and 2015 (*n* = 1). In addition, 124 sequences of H10N1 to H10N9 viruses from domestic and wild birds isolated from 1949 to 2015 were deposited in the databases. Amino acid differences in the HA1 of seal viruses compared to the avian counterparts studied herein are summarized in Table 1. Nine mutations—E82K, S113N, T165A, Q204K, N206S, Q220L, N236K, T238I, and M321V (H10 numbering corresponding to positions 91, 122, 171, 210, 212, 226, 242, 244, and 327 in H3 numbering)—were common in the HA1 of seal viruses, with a prevalence rate ranging from 57.7 to 100%. These positions are highly conserved in the European avian viruses, ranging from 84.7 to 100% (Table 1). None of these mutations was observed in the available sequences from mink (*n* = 2), pigs (*n* = 1), or humans (*n* = 4) (Supplementary Table S2). Except for M321V, the prevalence of these mutations in non-European H10Nx viruses ranged from 0 to 5.7% (Supplementary Table S2). All mutations except E82K and M321V reside in the HA head domain (Figure 1). E82K is located in the proximity of the stalk domain (adjacent to the head domain), and M321V is a part of the cleavage site. Twenty-five out of 26 seal viruses have ^318^PE(L/I)VQGR^324^|GLFGAIA (the cleavage site is after R324 and upstream of the GLFGAIA peptide) as the cleavage site motif, whereas avian H10Nx viruses possess the cleavage site motif ^318^PE(L/I/V)MQGR^324^|GLFGAIA. Mutations in position N236K and/or T238I resulted in absence of glycosylation at ^236^NIT^238^ (Figure 2A) due to lysine and/or isoleucine substitution ^236^KIT^238^ or ^236^KII^238^. The molecular weight of glycosylated wild-type H10N4 HA is higher than that of HA of N236K and T238I carrying viruses (Figure 2A). This *N*-glycosylation motif is highly conserved in avian viruses, where 118 out of 124 (95.9%) sequences contain this potential glycosylation site. On the monomer structure of HA, Q220L resides in the inner rim of the receptor binding pocket, whereas Q204K, N206S, N236K, and T238I are located parallel to each other near the 190-helix and 220-loop (Figure 1).

### 3.2. Rescue of Recombinant Viruses

The wild-type H10N4, H10N4 carrying HA from seal H10N7, and single mutants carrying unique mutations in positions E82K, S113N, Q204K, N206S, Q220L, N236K, T238I, or M321V, except T165A, were successfully rescued. All viruses were propagated in specific pathogen-free eggs, and the plaque titer was determined as described above.

### 3.3. Receptor Binding

Affinity to avian and mammalian SA receptors was compared using modified TRBCs and solid phase assay. Avian influenza viruses are able to bind to SA with α2,3 (avian-like) and α2,6 (mammalian-like) linkages. Therefore, the viruses were tested in an HA test with TRBCs, which carry both α2,3- and α2,6-SA, and modified TRBCs, which carry only the α2,6 human-type receptors. Results indicate that H10N4 virus recognized only the avian receptors and did not bind to the modified TRBCs. The seal H10N7 virus exhibited dual receptor affinity to both avian and mammalian receptors in TRBCs (Figure 2B,C). Q220L, the mutation at RBD, remarkably increased the affinity to mammalian-type receptors and completely abolished binding of H10N4 to avian-type receptors, thus making it similar to the human H1N1/PR8 (Figure 2C). Also, E82K and, to a lesser extent, T238I, M321V, S113N, and N206S increased the affinity of H10N4 to mammalian-type receptors (Figure 2C). Other viruses showed comparable affinity to the avian receptors.

### 3.4. Western Blot

Since M321V is very close to the HA cleavage site, the impact on cleavability was assessed by Western blot in MDCKII cells after single (6 hpi) or multiple (24 hpi) cycles with or without the addition of exogenous trypsin (Figure 2D). In the absence of trypsin, both viruses, the reverse-engineered H10N4 and H10N4 carrying M321V, were inefficiently cleaved (Figure 2D). Conversely, in the presence of trypsin, the HA of both viruses was cleaved into HA1 and HA2 subunits without obvious difference (Figure 2D).

### 3.5. Replication Kinetics

**Figure 3:**
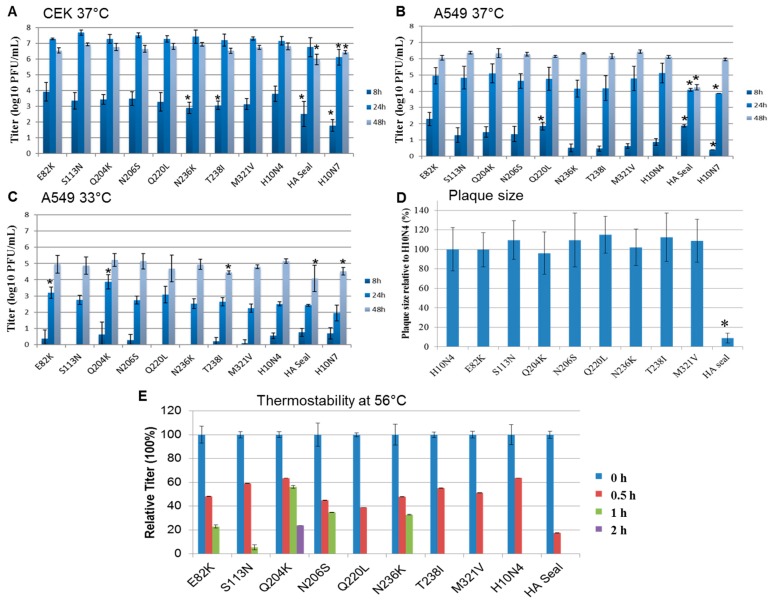
Replication kinetics and cell-to-cell spread of recombinant H10 viruses. Shown are the averages and standard deviations of replication kinetics of indicated viruses in (**A**) primary chicken embryo kidney cells at 37 °C and (**B**) human lung adenocarcinoma cells at 37 °C and (**C**) 33 °C at 8, 24, and 48 h postinfection. Cell-to-cell spread was assayed by measuring 50 to 100 plaques in MDCKII cells; (**D**) results are expressed as percentage of the average diameter of plaques induced by H10N4. Statistically significant values compared to the H10N4 virus are shown by asterisks. Thermostability of 10^5^ plaque-forming units (PFU) of each virus was determined after incubation at 56 °C for 4 h. The test was conducted in duplicate and repeated twice, and the virus titer was determined by plaque assay in MDCKII cells. All viruses were inactivated after 2 h; (**E**) shown are the relative titers at each indicated time point compared to titers before inactivation.

Viruses replicated in avian and mammalian cell lines. The temperature in the upper respiratory tract of birds is about 39 °C, which is higher than the temperature of the respiratory tract of mammals (about 33 °C). Therefore, replication kinetics of different mutants were studied in CEK at 37 °C and in A549 cells at 37 °C and 33 °C. All viruses replicated in CEK (Figure 3A) to at least 10-fold higher titers than in A549 at 37 °C (Figure 3B).

In CEK cells, mutations in HA1 did not significantly affect H10N4 virus replication at 24 and 48 h (Figure 3A). Replication of H10N7 and the recombinant expressing HA seal was significantly lower than that of the parental H10N4 (*p* < 0.002 and 0.03, respectively), where the titers of the seal virus H10N7 were 10- to 100-fold lower than those of the avian H10N4 virus. Also, at 8 hpi, N236K and T238I replicated at significantly lower levels than H10N4 virus (*p* < 0.004) (Figure 3A).

In A549 cells at 37 °C, at 8 hpi, H10N4 viruses carrying E82K, Q220L, or H10 from seal replicated to significantly higher levels than the H10N4 virus (*p* < 0.0008) and, interestingly, about 100 times higher than the seal H10N7 (*p* < 0.003) (Figure 3B). However, at 24 hpi, mutations did not affect the replication efficiency of H10N4 virus. At 24 hpi, HA-seal H10N4 virus and seal H10N7 virus replicated at significantly lower levels compared to the H10N4 virus (*p <* 0.02 and 0.007, respectively). At 48 hpi, all viruses except seal-HA H10N4 virus reached comparable replication titers (Figure 3B).

In A549 cells at 33 °C, replication of all viruses was reduced compared to their growth rate at 37 °C (Figure 3C). At 8 hpi, the titers were very low for all viruses and below the detection limit for mutants S113N, Q220L, and N236K (Figure 3C). Remarkably, E82K and Q204K significantly increased replication of H10N4 virus at 24 hpi (*p* < 0.002). All viruses replicated to approximately comparable levels at 48 hpi, where H10N4-T238I, HA seal, and H10N7 replicated at significantly lower titers than H10N4 (*p* < 0.04) (Figure 3C).

### 3.6. Cell-to-Cell Spread

To determine cell-to-cell spread, plaque assays using MDCKII cells were performed, and plaque sizes were measured and compared to wild-type H10N4. Results indicate that single mutations in HA1 did not significantly affect cell-to-cell spread. However, H10N4 virus carrying HA from seal virus produced very small plaques compared to other viruses (*p* < 0.00001) (Figure 3D). The seal H10N7 virus produced minute plaques, which could only be visualized under a microscope.

### 3.7. Heat Stability

Stability of AIV at high ambient temperature may facilitate the persistence or spread of the virus. Thus, it was important to investigate the impact of those unique mutations on heat stability. After 30 min at 56 °C, the titers of recombinant H10N4 viruses were reduced to 18 to 64% of their original titers (Figure 3E). Virus specifying H10 HA from seal was rapidly inactivated, while H10N4 viruses containing mutations N204K, N206S, and N236K and, to a lesser extent, E82K and S113N were infectious for at least 1 h. Only recombinant H10N4 virus with the N204K mutation retained infectivity for at least 2 h (Figure 3E). All viruses were inactivated after 2 h.

## 4. Discussion

While the mutation rate of AIVs in wild aquatic birds is low and considered to be mainly in a “stasis” form [1], interspecies transmission from wild birds to domestic birds or to mammals requires adaptive genetic changes to facilitate virus replication in these non-reservoir hosts [34]. Changes in receptor binding from avian to mammalian receptors are essential for efficient interspecies transmission in mammals or the emergence of pandemic viruses [35]. Seals may act as mixing vessels for the generation of pandemic viruses due to frequent infection by human and avian viruses [22,36,37,38,39,40]. Although controversial, some studies have shown that the respiratory tract of seals possesses both avian-like and human-like receptors [37], while others have confirmed only the presence of avian 2,3-SA in the lungs of seals [41]. In 2014, H10N7 viruses were isolated from seals on the northeastern coast of Europe during one of the largest outbreaks of AIV in sea mammals. All gene segments of this virus were closely related to avian influenza viruses, including H10N4, isolated from wild birds in Europe, suggesting direct transmission of one avian influenza virus to seals [18,19,22]. However, compared to the avian H10 viruses, the seal viruses carried unique mutations in HA1, mostly in the head region or close to the receptor binding domain. This could be due to the natural selection pressure of host species, which acts first on surface proteins [18]. Using reverse genetics, we successfully generated recombinant H10N4 viruses carrying full seal H10 or single mutations specific to the HA1 of seal H10N7 viruses isolated in the 2014 outbreak.

In contrast to avian H10N4 and H4N2 viruses, seal H10N7 virus bound to mammalian SA receptors but retained binding affinity to avian-type receptors. These results are also in accordance with previous reports, which described dual receptor affinity of avian H3N8 viruses isolated from New England harbor seals in 2011, in which the H3N8 affinity to avian 2,3-SA receptors was significantly higher than binding to 2,6-SA receptors [36,42,43,44]. Increased binding affinity to 2,6-SA receptors may be attributed to the Q220L mutation. This position resides directly in the RBD. H10N4 virus carrying Q220L bound effectively to 2,6-SA receptors, as did human H1N1 virus. It was previously described that double mutations Q226L and G228S (H3 numbering, which corresponds to 220 and 222 in H10 viruses) are essential for the adaptation of avian H2N2 and H3N2 subtypes in humans [45]. All seal viruses carry the avian G222 residue. Also, L220 has been implicated in the adaptation of equine H3N8 virus in dogs [46]. Therefore, this Q220L mutation that is present in a major fraction of seal H10N7 viruses may have been similarly important in their stepwise adaptation in seals. Nevertheless, all H10 viruses in this study replicated at lower levels in human lung cells than in avian cells, which may indicate poor adaptation to human cells. However, viruses carrying E82K, Q220L (with high affinity to 2,6-SA receptors), and HA from seal H10N7 virus produced relatively high titers in A549 cells after 8 h at 37 °C. Remarkably, N204K, N206S, and N236K and, to lesser extent, E82K and S113N increased thermostability of the virus, which may be important for efficient transmission between seals. It has been shown that some phenotypic traits of HA, such as human receptor binding preference and heat stability, are critical for airborne transmission of H5N1 AIV between ferrets [47,48]. Interestingly, the lack of glycosylation at the ^236^NIT^238^ motif due to mutations in position N236K and/or T238I increased affinity to human-type receptors and reduced H10N4 virus replication in CEK, suggesting a role in virus adaptation, as seen in several human H5N1 viruses [49,50]. The impact of these mutations in double, triple, or multiple combinations remains to be investigated. Beyond HA, other mutations, particularly in polymerase genes, may be additionally required for efficient replication in human cells [51]. It was also observed that both the seal isolate H10N7 and the “seal-HA H10N4” replicated at lower levels than recombinant avian H10N4 viruses in CEK cells, which may indicate a gradual loss of fitness for the original avian host. Moreover, in previous reports, mutations in the vicinity of the cleavage site of AIV affected cleavage activation of the virus and were correlated with increased virulence [52,53]. The seal H10N7 virus possesses M321V, which is located in the vicinity of the HA cleavage site. However, we could not observe any obvious difference in the cleavability of HA into HA1 and HA2 subunits in the presence or absence of trypsin. Whether this mutation affects the cleavability of the virus by other proteases (e.g., TMPRSS2, HAT) [54] remains to be investigated.

Altogether, although seal H10N7 virus retained strong binding affinity to avian-type receptors, the virus showed increased affinity to mammalian-type receptors, mainly due to the Q220L mutation in the receptor binding domain. The mutations analyzed appear to contribute to virus adaptation in seals/mammals and to a virus that can replicate and infect both avian and mammalian cells.

## Figures and Tables

**Table 1:** Prevalence of fixed mutations in seal viruses compared to other European H10Nx viruses.

Mutation		Avian Viruses	Number *n* = 124 (%)	Seal Viruses	Number *n* = 26 (%)	Substitution
H10 Numbering	H3 Numbering					
82	91	E	116 (93.5)	K	25 (96.2)	E82K
113	122	S	119 (96.0)	N	25 (96.2)	S113N
165 *	171	T	124 (100.0)	A	22 (84.6)	T165A
204	210	Q	124 (100.0)	K	22 (84.6)	Q204K
206	212	N	123 (99.2)	S	15 (57.7)	N206S
220	226	Q	124 (100)	L	17 (65.4)	Q220L
236 **	242	N	118 (95.9)	K	23 (88.5)	N236K
238 **	244	T	123 (99.2)	I	15 (57.7)	T238I
321 ***	327	M	105 (84.7)	V	25 (96.2)	M321V

* Recombinant virus with this mutation could not be rescued. ** Mutations in these positions resulted in deglycosylation of ^236^NIT^238^ to ^236^KIT^238^ or ^236^KII^238^. *** Mutation in this position changed the cleavage site from ^318^PE(L/I)MQGR^324^|GLFGAIA to ^318^PE(L/I)VQGR^324^|GLFGAIA.

## Supplementary Materials

The following are available online at http://www.mdpi.com/1999-4915/10/2/83/s1, Table S1: Acknowledgment of authors and originating and submitting laboratories of the sequences from GISAID’s EpiFlu database, Table S2: Prevalence of different mutations in mammalian and non-European H10Nx viruses.
