# Supplementary material for: Impact of Mutations in the Hemagglutinin of H10N7 Viruses Isolated from Seals on Virus Replication in Avian and Human Cells
Source: Viruses. 2018 Feb 14;10(2):83. doi: 10.3390/v10020083 (PMC5850390; doi:10.3390/v10020083)
Supplement: Supplementary file 1 [file viruses-10-00083-s001.pdf]

We acknowledge the authors, originating and submitting laboratories of the sequences from GISAID's EpiFlu™ Database on which this research is based. The list is detailed below.

All submitters of data may be contacted directly via the GISAID website ***www.gisaid.org***

Segment ID	Segment	Country	Collection date	Isolate name	Originating Lab	Submitting Lab	Authors
EPI178459	HA	Italy	1967-Jan-01	A/Turkey/Italy/928/1967		Istituto Zooprofilattico Sperimentale della Lor	
EPI169461	HA	United Kingd	1985-Jan-01	A/Fowl/Hampshire/PD378/1985	Veterinary Laboratories Agency, Weybridge	National Veterinary Institute	
EPI178543	HA	Italy	1966-Jan-01	A/Quail/Italy/1966		Istituto Zooprofilattico Sperimentale della Lor	
EPI278612	HA	Belgium	2009-Mar-21	A/Tadorna tadorna/Belgium/3441-P2/2009	Veterinary and Agrochemical Research Institute	Veterinary and Agrochemical Research Instit	
EPI372496	HA	Ukraine	2006-May-01	A/pied avocet/Ukraine/05848-NAMRU3/2006	U.S. Naval Medical Research Unit No.3	Centers for Disease Control and Prevention	Gerloff, Nancy; Simpson, Natosha; Jones, Joyce; Kis, Zoltan; Bahgat, Verina; Soliman,Atef; Elass
EPI169477	HA	Germany	1949-Jan-01	A/chicken/Germany/N/1949		National Veterinary Institute	
EPI774514	HA	Netherlands	2015-Apr-29	A/chicken/Netherlands/15007212/15	Central Veterinary Institute	Central Veterinary Institute	Bergervoet, Saskia; Heutink, Rene; Verschuren-Pritz, Sylvia; Harders, Frank; Bossers, Alex; Beere
EPI317614	HA	Germany	2007-Jan-01	A/Mallard/Germany/R2075/2007	Friedrich-Loeffler-Institut	Friedrich-Loeffler-Institut	Stech,O; Weber,S; Mettenleiter,TC; Stech,J
EPI178536	HA	Italy	2007-Jan-01	A/Duck/Italy/60772/2007		Istituto Zooprofilattico Sperimentale della Lor	
EPI178528	HA	Italy	2006-Jan-01	A/Duck/Italy/62330/2006		Istituto Zooprofilattico Sperimentale della Lor	
EPI178493	HA	Italy	2004-Jan-01	A/Duck/Italy/268302/2004		Istituto Zooprofilattico Sperimentale della Lor	
EPI174777	HA	Italy	2006-Jan-01	A/duck/Italy/73383/2006		Istituto Zooprofilattico Sperimentale della Lor	
EPI169500	HA	Belgium	1978-Jan-01	A/duck/Belgium/380/1978		Veterinary and Agrochemical Research Instit	
EPI541472	HA	Denmark	2011-Nov-14	A/mallard/Denmark/16109-4/2011-11-14	Technical University of Denmark	Technical University of Denmark	Hjulsager, Charlotte; Breum, Solvej; Trebbien, Ramona; Larsen, Lars E
EPI552756	HA	Netherlands	2012-Jan-14	A/mallard/Netherlands/1/2012	Erasmus Medical Center	Erasmus Medical Center	Bodewes, Rogier; Bestebroer, Theo M.; Van der Vries, Erhard; Verhagen, Josanne H.; Koopmans,
EPI552753	HA	Netherlands	2010-Nov-26	A/mallard/Netherlands/47/2010	Erasmus Medical Center	Erasmus Medical Center	Bodewes, Rogier; Bestebroer, Theo M.; Van der Vries, Erhard; Verhagen, Josanne H.; Koopmans,
EPI552752	HA	Netherlands	2010-Dec-03	A/mallard/Netherlands/50/2010	Erasmus Medical Center	Erasmus Medical Center	Bodewes, Rogier; Bestebroer, Theo M.; Van der Vries, Erhard; Verhagen, Josanne H.; Koopmans,
EPI552751	HA	Netherlands	2014-Feb-17	A/mallard/Netherlands/1/2014	Erasmus Medical Center	Erasmus Medical Center	Bodewes, Rogier; Bestebroer, Theo M.; Van der Vries, Erhard; Verhagen, Josanne H.; Koopmans,
EPI511811	HA	Italy	2007-Jan-01	A/mallard/Italy/4518/2007	Istituto Zooprofilattico Sperimentale Delle Venezie	Istituto Zooprofilattico Sperimentale Delle Ve	
EPI169485	HA	United Kingd	1985-Jan-01	A/Mallard/Gloucestershire/PD374/1985	Veterinary Laboratories Agency, Weybridge	National Veterinary Institute	
EPI332944	HA	France	2009-May-20	A/Anas_platyrhynchos/Camargue/091863/09	Station Biologique "Tour du Valat"	Institut Pasteur	VITTECOQ Marion, GRANDHOMME Viviane
EPI178551	HA	Italy	2006-Jan-01	A/Mallard/Italy/46341-12/2006		Istituto Zooprofilattico Sperimentale della Lor	
EPI278626	HA	Belgium	2009-Apr-01	A/Anas platyrhynchos/Belgium/4465-cls2/2009	Veterinary and Agrochemical Research Institute	Veterinary and Agrochemical Research Instit	
EPI223161	HA	Belgium	2009-Apr-01	A/Anas platyrhynchos/Belgium/4468/2009	Veterinary and Agrochemical Research Institute	Veterinary and Agrochemical Research Instit	
EPI223136	HA	Belgium	2009-Sep-21	A/Anas platyrhynchos/Belgium/09-2249/2008		Veterinary and Agrochemical Research Instit	
EPI541474	HA	Denmark	2014-Jul-01	A/harbor seal/Denmark/14-5061-1lu/2014-07	Technical University of Denmark	Technical University of Denmark	Krog, Jesper Schak; Hjulsager, Charlotte; Larsen, Lars E
EPI709090	HA	Sweden	2014-Sep-07	A/Seal/Sweden/SVA1412040224-SZ5634/H10N7/2014	Erasmus Medical Center	Erasmus Medical Center	Bodewes,Rogier
EPI709146	HA	Denmark	2014-Sep-30	A/harbor seal/DK/14-10580_L/2014	Erasmus Medical Center	Erasmus Medical Center	Bodewes,Rogier
EPI709145	HA	Denmark	2014-Sep-29	A/harbor seal/DK/14-10581_L/2014	Erasmus Medical Center	Erasmus Medical Center	Bodewes,Rogier
EPI709144	HA	Denmark	2014-Sep-26	A/harbor seal/DK/14-10585_L/2014	Erasmus Medical Center	Erasmus Medical Center	Bodewes,Rogier
EPI709143	HA	Denmark	2014-Aug-29	A/harbor seal/DK/14-8148_L/2014	Erasmus Medical Center	Erasmus Medical Center	Bodewes,Rogier
EPI709152	HA	Germany	2014-Nov-03	A/harbor seal/GER/FLI 2608_14/2014	Erasmus Medical Center	Erasmus Medical Center	Bodewes,Rogier
EPI709151	HA	Germany	2014-Nov-04	A/harbor seal/GER/FLI 2609_14/2014	Erasmus Medical Center	Erasmus Medical Center	Bodewes,Rogier
EPI709150	HA	Germany	2014-Nov-02	A/harbor seal/GER/FLI 2611_14/2014	Erasmus Medical Center	Erasmus Medical Center	Bodewes,Rogier
EPI709149	HA	Germany	2014-Nov-05	A/harbor seal/GER/FLI 2612_14/2014	Erasmus Medical Center	Erasmus Medical Center	Bodewes,Rogier
EPI709148	HA	Germany	2014-Nov-17	A/harbor seal/GER/FLI 2613_14/2014	Erasmus Medical Center	Erasmus Medical Center	Bodewes,Rogier
EPI709147	HA	Germany	2014-Oct-14	A/harbor seal/GER/PV20766_L/2014	Erasmus Medical Center	Erasmus Medical Center	Bodewes,Rogier
EPI709142	HA	Germany	2014-Oct-14	A/harbor seal/GER/PV20766_Tr/2014	Erasmus Medical Center	Erasmus Medical Center	Bodewes,Rogier
EPI709141	HA	Germany	2014-Oct-22	A/harbor seal/GER/PV20770_L/2014	Erasmus Medical Center	Erasmus Medical Center	Bodewes,Rogier
EPI709140	HA	Germany	2014-Oct-22	A/harbor seal/GER/PV20770_Tr/2014	Erasmus Medical Center	Erasmus Medical Center	Bodewes,Rogier
EPI709139	HA	Germany	2014-Oct-11	A/harbor seal/GER/PV20787_L/2014	Erasmus Medical Center	Erasmus Medical Center	Bodewes,Rogier
EPI709138	HA	Germany	2014-Oct-11	A/harbor seal/GER/PV20787_Tr/2014	Erasmus Medical Center	Erasmus Medical Center	Bodewes,Rogier
EPI709137	HA	Germany	2014-Oct-23	A/harbor seal/GER/PV20962_L/2014	Erasmus Medical Center	Erasmus Medical Center	Bodewes,Rogier
EPI709136	HA	Germany	2014-Oct-23	A/harbor seal/GER/PV20962_TrS/2014	Erasmus Medical Center	Erasmus Medical Center	Bodewes,Rogier
EPI709135	HA	Germany	2014-Dec-23	A/harbor seal/GER/PV20969_NS/2014	Erasmus Medical Center	Erasmus Medical Center	Bodewes,Rogier
EPI709134	HA	Germany	2014-Oct-07	A/harbor seal/GER/S1032_14_L/2014	Erasmus Medical Center	Erasmus Medical Center	Bodewes,Rogier
EPI709133	HA	Germany	2014-Oct-07	A/harbor seal/GER/S1040_14_L/2014	Erasmus Medical Center	Erasmus Medical Center	Bodewes,Rogier
EPI709132	HA	Germany	2014-Oct-07	A/harbor seal/GER/S1041_14_L/2014	Erasmus Medical Center	Erasmus Medical Center	Bodewes,Rogier
EPI709131	HA	Germany	2014-Oct-07	A/harbor seal/GER/S1042_14_L/2014	Erasmus Medical Center	Erasmus Medical Center	Bodewes,Rogier
EPI709130	HA	Germany	2014-Oct-10	A/harbor seal/GER/S1046_14_L/2014	Erasmus Medical Center	Erasmus Medical Center	Bodewes,Rogier
EPI709129	HA	Germany	2014-Oct-10	A/harbor seal/GER/S1047_14_L/2014	Erasmus Medical Center	Erasmus Medical Center	Bodewes,Rogier
EPI709128	HA	Germany	2014-Oct-07	A/harbor seal/GER/S1048_14_L/2014	Erasmus Medical Center	Erasmus Medical Center	Bodewes,Rogier
EPI709127	HA	Germany	2014-Sep-30	A/harbor seal/GER/S1050_14_L/2014	Erasmus Medical Center	Erasmus Medical Center	Bodewes,Rogier
EPI709126	HA	Germany	2014-Sep-30	A/harbor seal/GER/S1050_14_TS/2014	Erasmus Medical Center	Erasmus Medical Center	Bodewes,Rogier
EPI709125	HA	Germany	2014-Oct-01	A/harbor seal/GER/S1052_14_L/2014	Erasmus Medical Center	Erasmus Medical Center	Bodewes,Rogier
EPI709124	HA	Germany	2014-Oct-01	A/harbor seal/GER/S1052_14_TS/2014	Erasmus Medical Center	Erasmus Medical Center	Bodewes,Rogier
EPI709123	HA	Germany	2014-Oct-07	A/harbor seal/GER/S1054_14_L/2014	Erasmus Medical Center	Erasmus Medical Center	Bodewes,Rogier
EPI709122	HA	Germany	2014-Oct-07	A/harbor seal/GER/S1054_14_Tr/2014	Erasmus Medical Center	Erasmus Medical Center	Bodewes,Rogier
EPI709121	HA	Germany	2014-Oct-06	A/harbor seal/GER/S1055_14_L/2014	Erasmus Medical Center	Erasmus Medical Center	Bodewes,Rogier
EPI709120	HA	Germany	2014-Oct-06	A/harbor seal/GER/S1055_14_Tr/2014	Erasmus Medical Center	Erasmus Medical Center	Bodewes,Rogier
EPI709119	HA	Germany	2014-Oct-14	A/harbor seal/GER/S1070_14_L/2014	Erasmus Medical Center	Erasmus Medical Center	Bodewes,Rogier
EPI709118	HA	Germany	2014-Oct-14	A/harbor seal/GER/S1070_14_ThS/2014	Erasmus Medical Center	Erasmus Medical Center	Bodewes,Rogier
EPI709117	HA	Germany	2014-Oct-14	A/harbor seal/GER/S1070_14_Tr/2014	Erasmus Medical Center	Erasmus Medical Center	Bodewes,Rogier
EPI709116	HA	Germany	2014-Oct-14	A/harbor seal/GER/S1071_14_L/2014	Erasmus Medical Center	Erasmus Medical Center	Bodewes,Rogier
EPI709115	HA	Germany	2014-Oct-14	A/harbor seal/GER/S1071_14_ThS/2014	Erasmus Medical Center	Erasmus Medical Center	Bodewes,Rogier
EPI709114	HA	Germany	2014-Oct-14	A/harbor seal/GER/S1071_14_TrS/2014	Erasmus Medical Center	Erasmus Medical Center	Bodewes,Rogier

Supplementary Table S2: Prevalence of different mutations in mammalian and non-European H10Nx viruses

Residue		European Avian H10-viruses	Seal H10N7-viruses	Mammal-viruses (n=7)*		Non-European Avian Viruses (n=842)	
H10-Numbering	H3-Numbering			Avian-like	Seal-like	Avian-like	Seal-like
82	91	E	K	**E: 5(71.4%)**	K: 2 (28.6%)**	**E: 592(70.3%)**<br>D: 244(29%)<br>N: 1 (0.1%)	0
113	122	S	N	**S: 7(100%)**	0	**S: 809(96.1%)**<br>G: 14(1.7%)	N: 13(1.5%)
165	171	T	A	**T: 7(100%)**	0	**T: 834(99%)**<br>I/K: 7(0.9%)	A: 1(0.1%)
204	210	Q	K	**Q: 4(57.1%)**<br>R: 3(42.9%)	0	**E: 779(92.5%)**<br>R: 62(7.4%)<br>H: 1(0.1%)	0
206	212	N	S	**N: 7(100%)**	0	**N: 794(94.3%)**	S: 48(5.7%)
220	226	Q	L	**Q: 7(100%)**	0	**Q: 842(100%)**	0
236	242	N	K	**N: 6(85.7%)**<br>S: 14.3%)	0	**N: 835(99.2%)**<br>S: 6(0.7%)<br>D: 1(0.1%)	0
238	244	T	I	**T: 7(100%)**	0	**T: 841(99.9%)**	I: 1(0.1%)
321	327	M	V	**M: 2(28.6%)**<br>I: 5(71.4%)	0	**M: 154(18.3%)**<br>I: 74(8.8%)<br>A: 45(5.3%)<br>F: 1(0.1%)	V: 568(67.5%)

* Seven available sequences of H10Nx in mammals were retrieved: swine (one H10N5 from China), mink (two H10N4 from Sweden) and humans (four H10N8 from China). Two Australian H10N7 in humans were deposited in the GenBank, however only aa in positions 368 to 532 are available. Therefore, they were not included in the current analysis.

** positive samples were detected in mink.

Written in bold are those sequences with avian-like signatures as found in European H10Nx viruses.
